# Danggui Buxue Decoction exerts its therapeutic effect on rheumatoid arthritis through the inhibition of Wnt/β-catenin signaling pathway

**DOI:** 10.1186/s13018-023-04439-4

**Published:** 2023-12-09

**Authors:** Xin Jiang, Yanxin He, Ying Zhao, Zhi Pan, Yinghang Wang

**Affiliations:** 1 College of Integrative Medicine, Changchun University of Chinese Medicine, Changchun, Jilin China; 2 Jilin Ginseng Academy, Changchun University of Chinese Medicine, Changchun, Jilin China; 3 The Affiliated Hospital to Changchun University of Chinese Medicine, Changchun, Jilin China

**Keywords:** Danggui Buxue Decoction, Rheumatoid arthritis, Human fibroblast-like synoviocytes cells, Pro-inflammatory factors, Wnt/β-catenin signaling pathway

## Abstract

**Background:**

Danggui Buxue Decoction (DBD) is a traditional Chinese medicine prescription, which has the functions of benefiting Qi, generating blood and regulating the immune system. At present, various clinical reports suggest that DBD has some efficacy in Rheumatoid arthritis (RA), but its mechanism of action is still unclear. Thus, the present study explored mechanism of this preparation on RA.

**Methods:**

The effect of DBD was evaluated by tumor necrosis factor (TNF)-α-induced Human fibroblast-like synoviocyte of rheumatoid arthritis (HFLS-RA) cell model and collagen-induced arthritis (CIA) rat model, respectively. Inflammatory factors including TNF-ɑ, IL-1β, IL-6 and IL-10 in the culture supernatants or rat serum were measured using ELISA. The related indexes including fur luster, mental state and activity of rat and the symptoms including swelling and deformation of toes and ankles were also measured.

**Results:**

In vitro results showed that DBD cannot only inhibit the proliferation of HFLS-RA cells but also reduce the levels of pro-inflammatory factors while increasing the level of anti-inflammatory factors. Similar results were obtained from in vivo experiments. Rats receiving DBD showed a decrease in the severity of rheumatoid arthritis in rat models. Moreover, the protein levels of c-myc and β-catenin decreased significantly, while the protein level of SFRP4 increased, which indicated that DBD might inhibit the inflammatory reaction by regulating Wnt/β-catenin signaling pathway, thus alleviating the symptoms of RA.

**Conclusion:**

Our findings not only provide insights for understanding the molecular mechanism of DBD in treating RA, but also provide the theoretical basis for further clinical prevention and treatment.

## Introduction

Rheumatoid arthritis (RA) is a common clinical autoimmune disease that is characterized by chronic inflammation of the synovium surrounding a joint. Ultimately, such inflammation will cause irreversible damage to the joints of these patients and seriously affect their quality of life [1, 2]. The Wnt/β-catenin signaling pathway is closely related to the pathogenesis of RA, and the Wnt/β-catenin signaling pathway is overactivated in RA patients. Secretory frizzled-related proteins (SFRPS) are soluble proteins that not only act as antagonists but can also activate the Wnt/β-catenin signaling pathway. The SFRP4 gene can inhibit the Wnt/β-catenin signaling pathway and prevent synovial hyperplasia [3]. C-myc is an important downstream effector gene of the Wnt/β-catenin pathway and is directly related to the abnormal synovial hyperplasia of RA. The etiology of RA remains unclear, and the majority of patients require long-term medication after diagnosis. The side effects of long-term medication and drug resistance are serious problems and require urgent attention.

Danggui Buxue Decoction (DBD) is a representative prescription for benefiting Qi and activating blood circulation, which is composed of two traditional Chinese medicines, angelica and astragalus. Modern pharmacological research has found that DBD plays a beneficial role in regulating immunity and the blood system, enhancing estrogen-like activity, and exhibits antioxidation, anti-aging, and anti-inflammatory properties [4]. According to previous relevant studies, DBD contains sorts of biological active ingredients including astragaloside IV, ferulic acid, calycosin, formononetin, total flavonoids, total saponins, and total polysaccharides [5]. Astragaloside IV and ferulic acid have been demonstrated to attenuate the inflammatory reaction via inhibition of Wnt/β-catenin pathway [6, 7]. Calycosin can mediate inflammatory response [8], while in a previous in vivo study, administration of formononetin also had anti-inflammatory effect [9]. Consequently, DBD represents a promising new direction for the prevention and treatment of RA.

In this study, we combined a range of in vitro and in vivo experiments to explore the relationship between DBD and RA and to study the effects of DBD on RA and the mechanisms involved may be related to the inhibition of Wnt/β-catenin signaling pathway. Consequently, the aim of our present investigation is to explore new options for preventing and treating RA and to provide a theoretical and experimental basis for the clinical application of DBD.

## Materials and methods

### Reagents and materials

Freund's complete adjuvant and Thiazolyl blue tetrazolium bromide (MTT) was purchased from Sigma; Cell Counting Kit-8(CCK-8) were purchased from Beyotime; DMEM high glucose culture medium was purchased from Hyclone company (United States); recombinant human tumor necrosis factor (TNF)-α was obtained from Peprotech (United States); neonatal bovine serum and trypsin were obtained from GIBCO (United States); and 5× protein sample buffer was obtained from Novex (United States). Bovine type II collagen was purchased from Xinbosheng Biotechnology Co., Ltd.; 4% polyformaldehyde, TEMED, Dimethyl sulfoxide (DMSO) and Ethylenediaminetetraacetic acid (EDTA) were purchased from Beijing Dingguo Changsheng Biotechnology Co., Ltd; SDS, β-actin and SFRP4 were purchased from Biotoped; and a BCA kit, PMSF, RIPA lysate, and skimmed milk powder were obtained from Shanghai Biyuntian Biotechnology Co., Ltd. Protein Marker (10–180 kDa) was obtained from Beijing Solarbio Science and Technology Co., Ltd.; β-Catenin and c-myc were obtained from CST. We also purchased a range of ELISA kits to detect rat IL-1, rat IL-4, rat IL-6, and rat IL-10, and a range of ELISA kits to detect human IL-1β, human IL-6, human IL-10, and human TNF-α; these ELISA kits were purchased from Shanghai Langton Biotechnology Co., Ltd.

### Animal and cell culture

We obtained 80 Specific-Pathogen-Free Wistar rats (> 220 g; 40 males and 40 females). The rats were held in separate cages and acclimatized for one week prior to experimentation; during this time, the rats were given ad libitum access to normal rat food and distilled water. The photoperiod was set to 12L:12D, the room temperature was 22 °C, and relative humidity was 50–65%.

Human fibroblast-like synoviocytes of rheumatoid arthritis (HFLS-RA) cells (Beijing Beina Chuanglian Biotechnology Research Institute) were cultured in an incubator at a constant temperature of 37 °C in high humidity and 5% CO_2_. Cells were cultured in DMEM high glucose medium with 20% FBS. The cells were digested with 0.25% trypsin when they reached 80–90% confluency, and a cell suspension was created for further culture. All cells used in the subsequent experiments were obtained from passages 3–7.

### Preparation of DBD decoction

Astragalus membranaceus and Angelica (Tongrentang pharmacy, Beijing, identified by the school’s Chinese medicine identification teacher) were weighed and placed into a beaker in a ratio of 5:1. Distilled water was then added, and the materials were allowed to soak for 1 h. The decoction was then performed (When decocting, first fire and then light the fire). The solution produced was then filtered and decocted for a second time. Finally, the two decoctions were combined and concentrated in a constant temperature water bath to obtain a final concentration of 1.296 g of crude drug/mL; this represented a high concentration of DBD in water. We also prepared medium and low concentrations of DBD in water: 0.648 g crude drug/mL and 0.324 g crude drug/mL, respectively. These three decoctions were then sealed and stored in the dark at 4 °C.

### The preparation of drug-containing serum of DBD

Wistar rats were randomly divided into five groups: a blank group, a tripterygium glycoside group (8.75 mg/kg), a DBD low dose group (3.24 g/kg), a DBD medium dose group (6.48 g/kg), and a DBD high dose group (12.96 g/kg). Rats in each group were given different concentrations of DBD water decoction by gavage at a dose of 1 mL/100 g; rats in the blank group were given the same volume of distilled water once a day. One hour after administration, on the morning of the fourth day, we injected 20% urethane into the abdominal aorta of anaesthetized rats at a dose of 1 g/kg in order to take blood samples. All blood samples were left at room temperature for 2 h and then centrifuged for 10 min at 3000 rpm. After centrifugation, the supernatant was gently aspirated and placed into a clean microcentrifuge tube; this provided us with samples of the drug-containing serum. The drug-containing serum from rats in the same experimental group was mixed; this allowed us to create different groups of serum drugs. The serum was inactivated in a constant temperature water bath at 56 °C for 30 min and a microporous filter membrane (0.22 μm) was used to sterilize the sera. Finally, sera were sealed and frozen in a refrigerator at -20 °C to await subsequent experiments.

### Cell proliferation assays

MTT and CCK-8 detection of the effect of drug-containing serum on the proliferation of HFLS-RA cells. HFLS-RA cells were digested and made into a suspension. The cell density was then adjusted to 1** × **10^4^/mL; we then used 100 μL/well to inoculate a 96-well plate. Each experimental group was allocated with 8 multiple wells; there were 6 groups in total. Once the 96-well plates had been placed in an incubator for 4 h, the culture medium was carefully discarded, and each group of cells was treated with the appropriate drug, as shown in Table 1. After that, the cells were cultured for 24 h and 48 h. For MTT assay, we then added 20 μL MTT to the culture and cultured it for 4 h at 37 °C. Subsequently, we discarded the supernatant and added DMSO to each well. The optical density (OD) was measured at a wavelength of 490 nm. For CCK-8 assay, we added 10 μL CCK-8 to the culture and cultured it for 1 h at 37 °C. The OD was measured at a wavelength of 450 nm. Cell proliferation inhibition rate calculation formula: (Model group – DBD groups)/(Model group – Blank group) × 100%.

**Table 1:** Drug components in each group

Group	Component
Blank group	Serum from rats in the 10% blank group
Model group	Serum from rats in the 10% blank group + 20 ng/mL TNF-α
Tripterygium glycoside group	Serum from rats in the 10% tripterygium glycoside group + 20 ng/mL TNF-α
DBD low dose group	Serum from rats in the 10% DBD low dose group + 20 ng/mL TNF-α
DBD medium dose group	Serum from rats in the 10% DBD medium dose group + 20 ng/mL TNF-α
DBD high dose group	Serum from rats in the 10% DBD high dose group + 20 ng/mL TNF-α

### Detection the of TNF-α, IL-1β, IL-6 and IL-10 by ELISA

When the HFLS-RA cells reached 80% confluency, the cells were divided into different groups and stimulated with different medicinal regimens, as described in Table 1. Cells were then cultured for 24 h, after which, the supernatant was removed and stored at 4 °C. The levels of TNF-α, IL-1β, IL-6 and IL-10 in the supernatant were then detected using appropriate ELISA kits according to the manufacturer’s instructions. The level of TNF-α, IL-1β, IL-6 and IL-10 in the supernatant was determined as same as above described.

### Establishment of collagen-induced arthritis (CIA) rat model

We prepared a rat model of CIA with bovine type II collagen, as described in the previous literature, but with a few modifications [10]. Briefly, we prepared bovine type II collagen emulsion at a concentration of 1 g/L by emulsifying a mixture of glacial acetic acid solution and bovine type II collagen in an aseptic environment. We established a blank group and allocated the CIA rats into different groups: a model group, a Tripterygium glycoside group, a DBD high group, a DBD medium group, and a DBD low dose group. Wistar rats were immunized for the first time one week after adaptive feeding. When immunized for the first time, the rats were injected with 0.2 mL of type II collagen emulsion into the toe, tail root (1.5–2.0 cm from tail root), or subcutaneously, depending on the dosage. One week after the first immunization, we used the same method to strengthen the second immunization response but with a dose of 0.1 mL of type II collagen emulsion. The rats were divided into two groups and then, treated with drugs on the 12th day. Each group was administered with DBD decoction by gavage according at a dose of 1 mL/100 g; the blank group and the model group were given an equivalent volume of distilled water. The Tripterygium glycoside group was given Tripterygium glycoside suspension by gavage for 28 days at a dose of 8.75 mg/kg. During this time, we observed the general condition of each rat (mental state, appetite, and fur color). We also recorded any evidence of joint swelling. We made efforts to measure the rate of any joint swelling. At the beginning of the experiment, we recorded the peripheral diameter of the right ankle joint of each rat; we then re-measured this area once a day before the first immunization; these measurements were used as a baseline. Following the first immunization, we measured the right ankle joint once a week. We then calculated the swelling rate as follows: Swelling rate = (the peri-ankle diameter after inflammation—peri-ankle diameter before inflammation)/peri-ankle diameter before inflammation × 100%. We also calculated the arthritis index (AI) for each joint, using the following scoring criteria: no swelling of joint, 0 points; slight swelling of the small toe joint, 1 point; swelling of toe joint and toe, 2 points; swelling of the foot below ankle joint, 3 points; swelling of all feet including the ankle joints, 4 points. According to these scoring criteria, we scored the four toes of each rat and created an overall score by adding the score for each foot; the maximum score was 16 points. An AI value greater than 4 points indicated successful CIA modeling [11]. Paw swelling and arthritis scores are used to evaluate the anti-arthritic effects of drugs [12].

### Hematoxylin–eosin (H&E) staining to observe the morphological changes of ankle joint

Ankle joint tissues were first fixed with paraformaldehyde, decalcified, dehydrated, cleared and embedded in paraffin wax. Thin Sects. (4–6 microns) were then prepared, unfolded in water, baked and placed into a fixed frame on a slide for 10 h at 62 °C. For staining, sections were baked, dewaxed, stained in hematoxylin and eosin, dehydrated, fixed onto glass slides and sealed. Five visual fields were randomly selected and assessed for immunoreactive areas at × 200 magnification using a light microscope.

### Western blot analysis of β-Catenin, SFRP4 and c-myc in the synovium

The synovial tissue protein was extracted, and the protein content was determined by the BCA protein detection kit. Protein concentration was quantified with the Easy Protein Quantitative Kit. Protein was subjected to 12% sodium dodecyl sulfate–polyacrylamide gel electrophoresis and then, transferred onto poly-vinylidene fluoride membranes. Membranes were incubated with primary antibodies such as rabbit polyclonal antibody against β-actin (1:1000), rabbit polyclonal antibody against β-catenin (1:5000), and rabbit polyclonal antibody against sfrp4 (1:500) and rabbit monoclonal antibody against c-myc (1:1000) overnight at 4 °C. Beta-actin was used as an internal reference. The next day, the membranes were washed with PBST and then incubated with horseradish peroxidase labeled anti-rabbit IgG (1:5000, Bioworld company) for 1 h at room temperature. Protein bands were visualized using a chemiluminescent imaging system.

### Statistical analysis

All experimental data were processed and analyzed by SPSS version 13.0 software (SPSS Inc., Chicago, IL). Data were expressed as means ± standard deviation. Differences between groups were tested for significance by one-way analysis of variance (ANOVA). *P* < 0.05 was considered statistically significant.

## Results

### The effect of serum-containing DBD on the proliferation of HFLS-RA cells

**Fig. 1:**
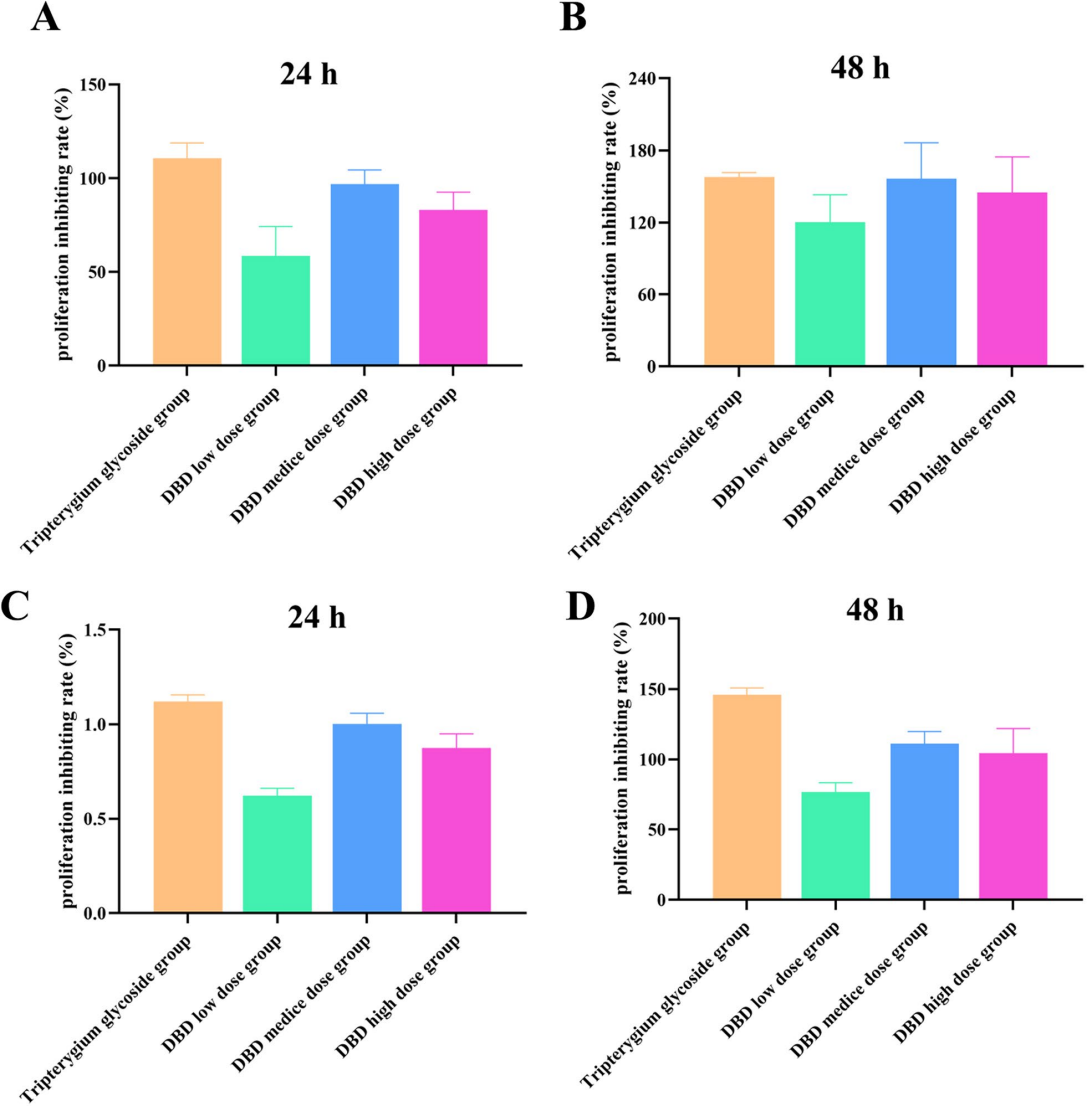
The effect of DBD on the viability of HFLS-RA cells with or without TNF-α stimulation. **A**. MTT assay: HFLS-RA was incubated with DBD for 24 h. **B**. MTT assay: HFLS-RA was incubated with DBD for 48 h. **C**. CCK-8 assay: HFLS-RA was incubated with DBD for 24 h. **D**. CCK-8 assay: HFLS-RA was incubated with DBD for 48 h. Cell proliferation was measured by MTT and CCK-8 assay

As shown in Fig. 1, MTT and CCK-8 assays showed that the proliferation of HFLS-RA cells in the DBD groups was obviously inhibited after 24 h and 48 h.

### The effect of DBD on the level of TNF-α, IL-1β, IL-6, and IL-10 in cell supernatants

**Fig. 2:**
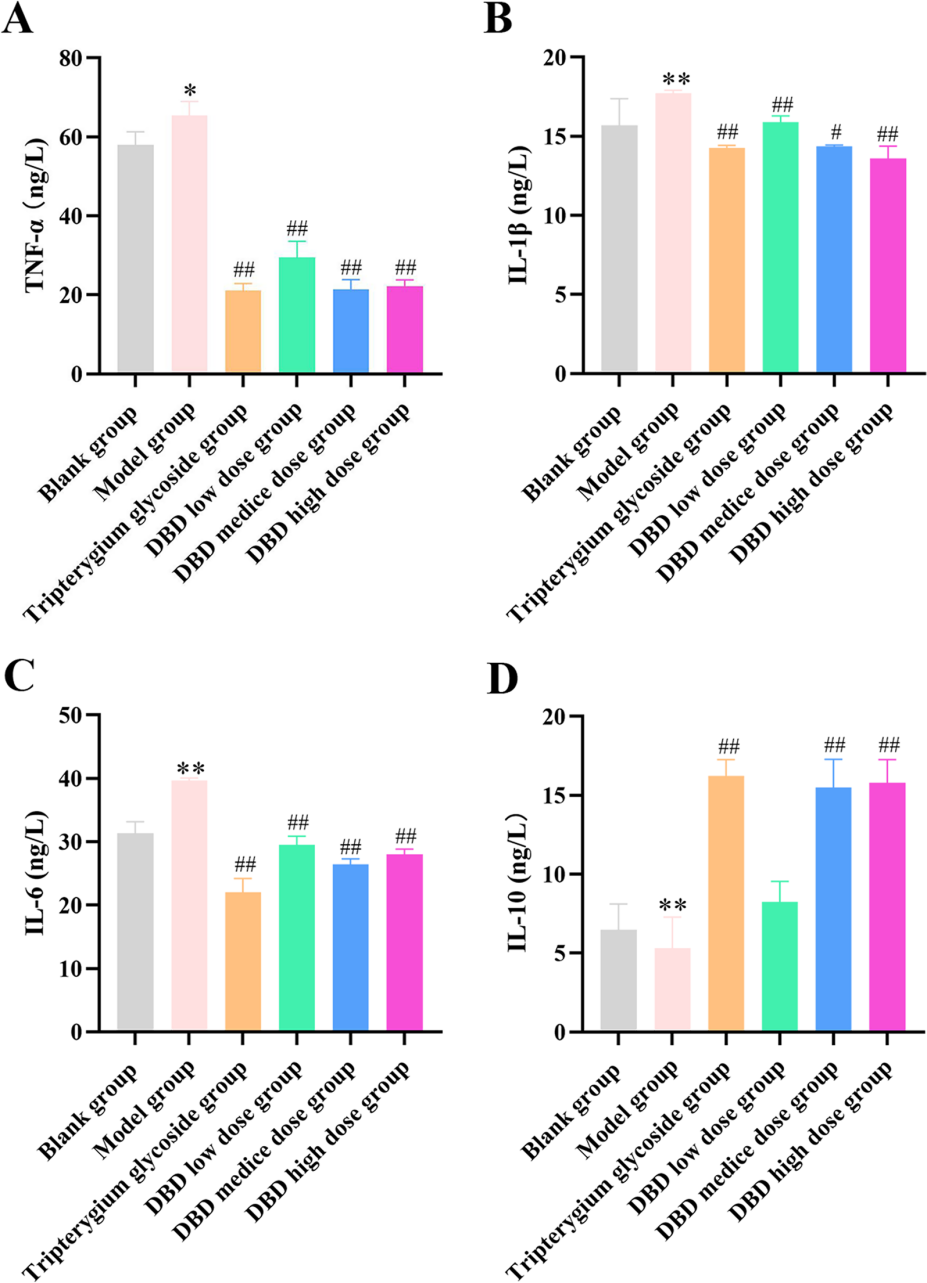
Levels of TNF-α, IL-1β, IL-6 and IL-10 in cell supernatant. **A**. TNF-α. **B**. IL-1β. **C**. IL-6. **D**. IL-10. Compared with the blank group, *P < 0.05, **P < 0.01; compared with the model group, ^#^*P* < 0.05, ^##^*P* < 0.01. Mean ± standard deviation, *n* = 6

The levels of TNF-α, IL-1β, and IL-6 in the supernatant were detected by ELISA. This result showed that compared with the blank group, the levels of TNF-α, IL-1β, and IL-6 in the supernatant from model group cells were significantly increased, while the levels of IL-10 were significantly decreased. Furthermore, compared with the model group, the levels of TNF-α, IL-1β and IL-6 in the supernatant of the cells that treated with Tripterygium polycoside and high dose DBD were significantly reduced, while the levels of IL-10 were significantly elevated (Fig. 2). These results indicated that DBD can reduce the levels of pro-inflammatory factors such as TNF-α, IL-1β and IL-6 while increasing the level of anti-inflammatory factors including IL-10, which relives inflammatory active.

### General observations

**Fig. 3:**
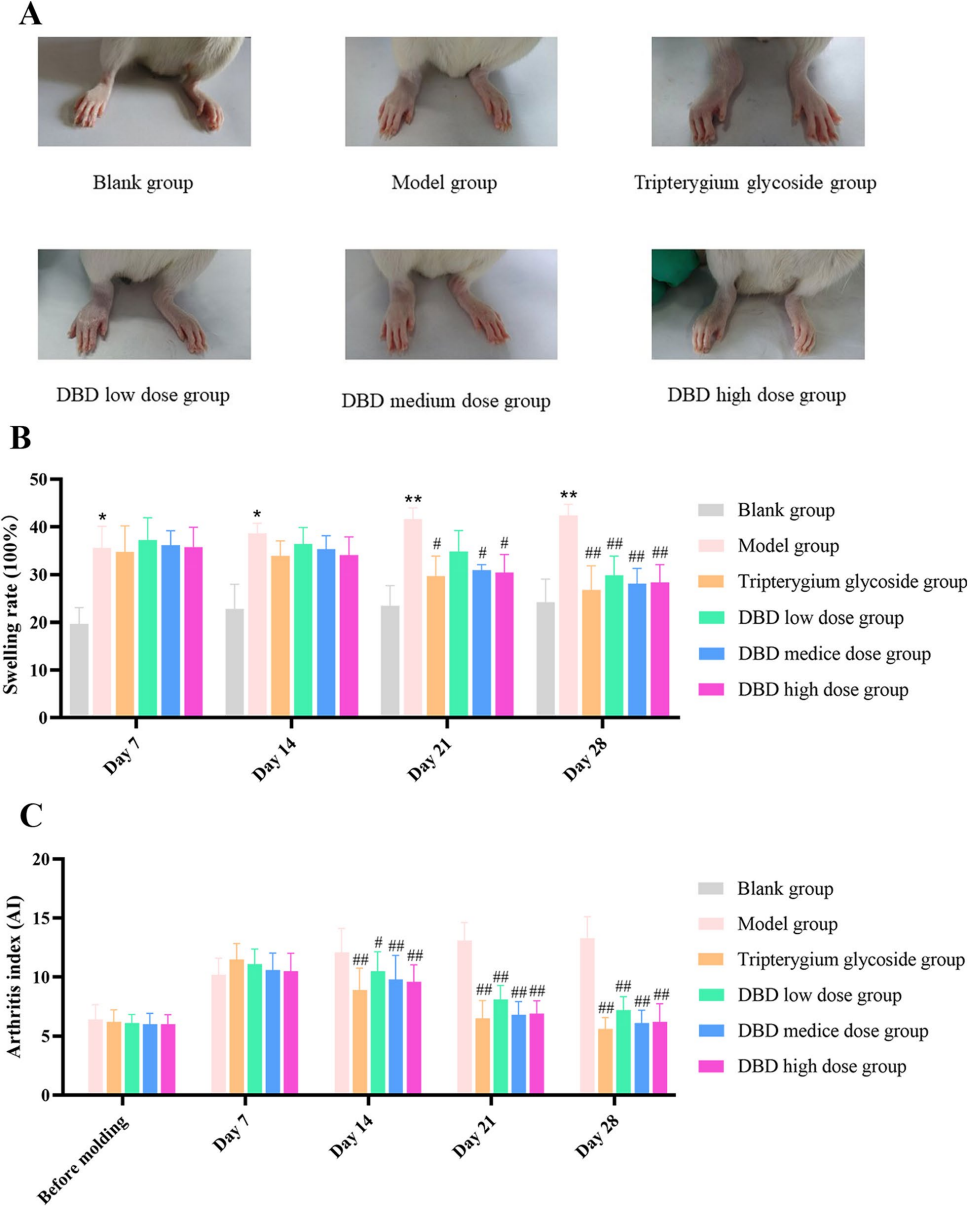
The effect of DBD on reducing the swell of the foot**. A**. Representative picture of joint swelling in CIA rats. **B**. Comparison of joint swelling rate in rats from each group during treatment. Data are means ± SD of 6 rats per group. Compared with the blank group, *P < 0.05, **P < 0.01; compared with the model group, ^#^*P* < 0.05, ^##^*P* < 0.01. **C**. Comparison of the AI of rats in each group during treatment. Data are means ± SD of 6 rats per group. Compared with the model group, ^#^*P* < 0.05, ^##^*P* < 0.01

In order to further determine the effect of DBD on RA in vivo, CIA rat models were constructed using bovine type II collagen and then, evaluated by general observations. Firstly, fur luster, mental state and activity of rat were observed. We found that the rats in the blank group were in a normal mental state, with shiny fur and active activities, and there were no obvious abnormalities. With the prolongation of feeding time, the weight of rats in the blank group gradually increased. Compared with the blank group, CIA rats showed mental depression, loss of appetite, fur luster and limited activity and swelling and deformation of toes and ankles to different degrees, although none of the animals in this group died. At the same time, we treated CIA rats with different group components from Table 1 and found that compared with the model group, the symptoms including swelling and deformation of toes and ankles were alleviated after administration with the Tripterygium glycoside group and the DBD low, medium, and high dose (Fig. 3A). The swelling rates of the rat ankles were also detected. As shown in Fig. 3B, the swelling rates of the rat ankles in the model group were significantly higher than those in the blank group. Compared with the model group, the swelling rates of ankles in the Tripterygium glycoside group, and the DBD low, medium, and high dose group, were significantly lower. The result of an assessment based on an AI showed that compared with the model group, the AI of rats after treatment in each DBD dose group decreased significantly, especially in the Tripterygium glycoside group, the DBD high and medium dose groups (Fig. 3C).

### Morphological changes in the ankle

**Fig. 4:**
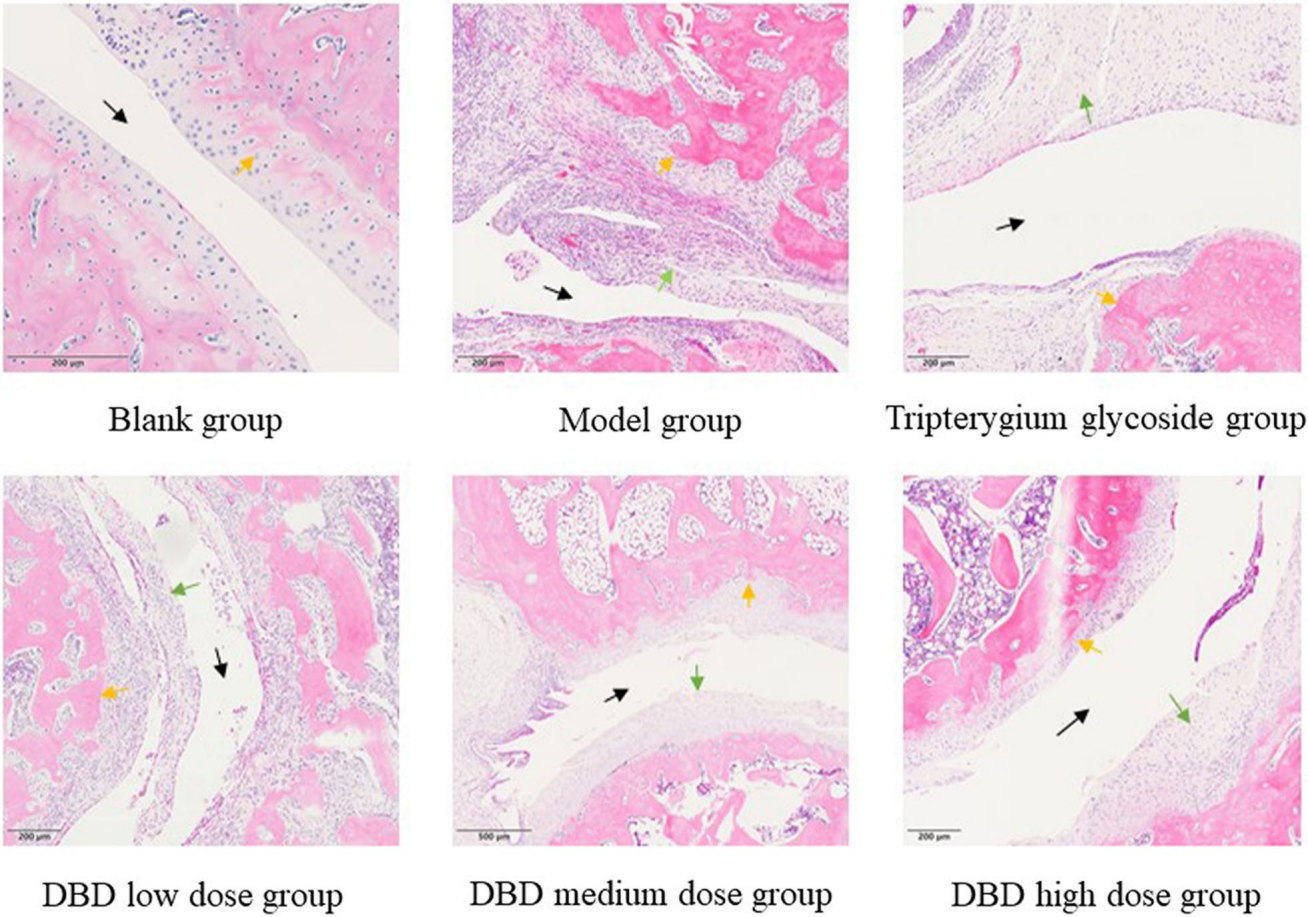
Effect of DBD on joint tissue in experimental rats as determined by H&E staining (200 × magnification). Black arrow: joint cavity; orange arrow: articular cartilage; green arrow: synovial tissue

HE staining showed that the morphology and structure of the ankle were normal in the blank group because there was no tissue necrosis, infiltration of inflammatory cells, or interstitial edema in rat ankles. Compared with the blank group, the model group showed severe hyperplasia, infiltration of inflammatory cells, synovial angioedema and pannus formation. While compared with the model group, the swelling and congestion of the synovium and the proliferation of synovium blood vessels in Tripterygium glycosides group and the DBD low, medium and high dose groups were improved to varying degrees, and there was no obvious inflammatory cell infiltration or fibrosis (Fig. 4).

### The effect of DBD on the level of IL-4, IL-1β, IL-6, and IL-10 in vivo

**Fig. 5:**
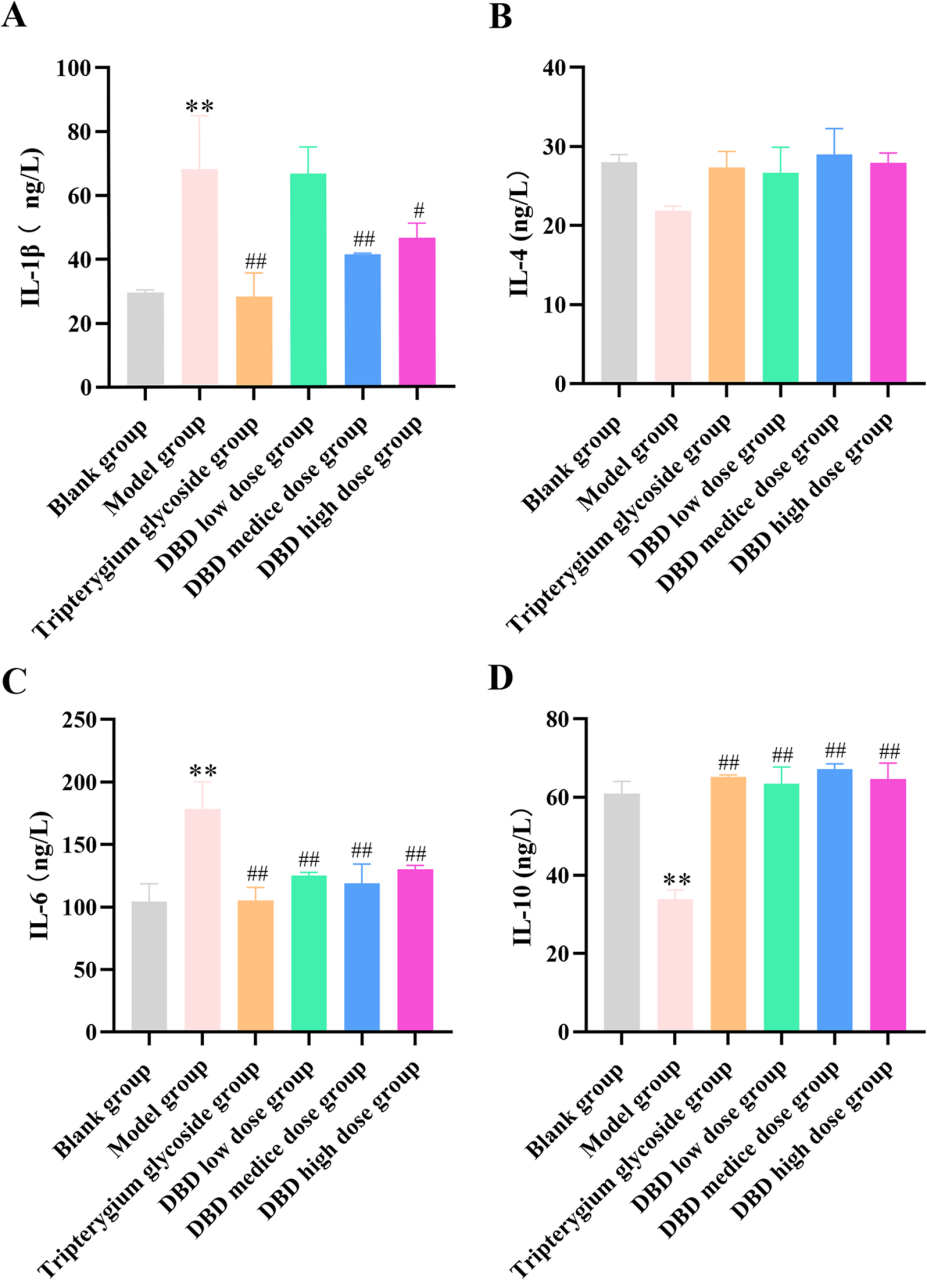
Serum levels of IL-1β, IL-4, IL-6 and IL-10 in rats from different treatment groups (mean ± standard deviation, *n* = 6). **A**. IL-1β. **B**. IL-4. **C**. IL-6. **D**. IL-10. Compared with the blank group, *P < 0.05, **P < 0.01; compared with the model group, ^#^*P* < 0.05, ^##^*P* < 0.01

According to ELISA, compared with the normal group, the levels of IL-1β and IL-6 in the model group increased significantly, while the levels of IL-4 and IL-10 decreased significantly. Compared with the serum of the model group, DBD and Tripterygium glycoside significantly attenuated the levels of IL-1β and IL-6 in CIA rat serum, while the levels of IL-4 and IL-10 were raised (Fig. 5).

### The effect of DBD on Wnt/β-catenin signaling pathway

**Fig. 6:**
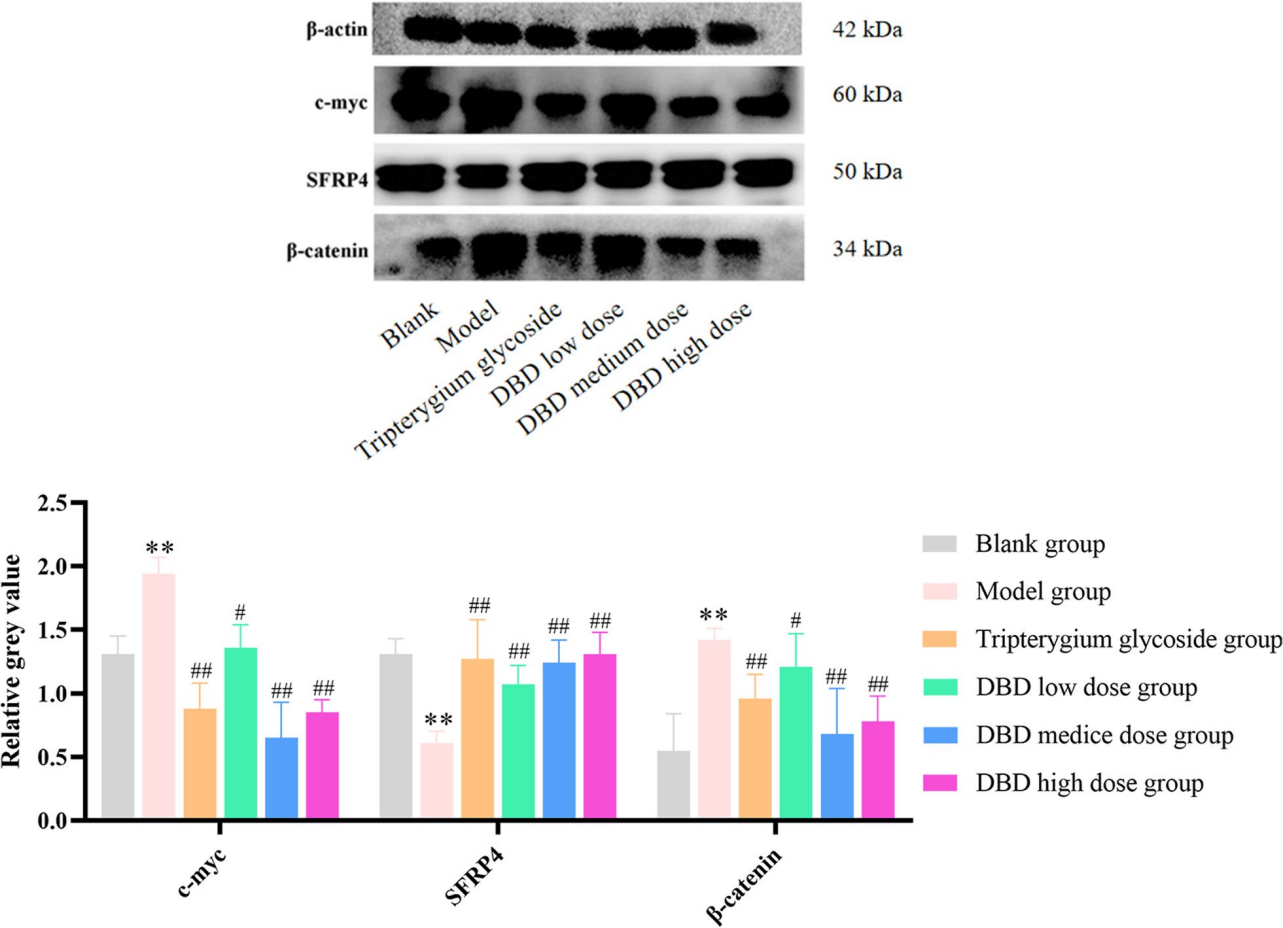
The effects of DBD on the levels of β-catenin, SFRP4 and c-myc protein in the synovium of experimental rats. Compared with blank group, *P < 0.05, **P < 0.01; compared with model group, ^#^*P* < 0.05, ^##^*P* < 0.01

The canonical Wnt/β-catenin pathway has been implicated in the pathogenesis of RA; therefore, western blotting was used to detect Wnt/β-catenin signal pathways–related protein expression. As shown in Fig. 6, compared with the blank group, the levels of c-myc and β-catenin protein in the synovium of the model group were significantly increased, and the levels of SFRP4 protein were decreased. Compared with the model group, the levels of c-myc and β-catenin protein in the tripterygium glycoside group and the DBD low, medium and high dose groups were significantly decreased, while the level of SFRP4 protein was increased. These results suggested that DBD can inhibit Wnt/β-catenin signaling pathway.

## Discussion

According to the clinical manifestations and developmental process of RA, traditional Chinese medicine (TCM) ascribes RA to the category of “Bi syndrome.” The main causes of Bi syndrome are external evil, disharmony of Ying and Wei, and deficiency of the prime body. At present, most experts in TCM have a certain consensus of opinion with regard to the treatment of arthralgia. For example, Zhao stressed that the treatment of arthralgia should be based on the principle of strengthening the body and eliminating pathogenic factors and the method of Warming Yang and promoting arthralgia [13]. Astragalus, as a TCM, is able to strengthen Qi, as well as promoting water and swelling, promoting body fluid and blood circulation, promoting stagnation and arthralgia, supporting toxin and purulent discharge, and accumulating sore and generating muscle. Therefore, Astragalus is commonly used in the clinical treatment of arthralgia caused by deficiency [14]. As an important medicine in blood, Angelica cannot only replenish blood but can also provide a number of beneficial effects. For example, Angelica can replenish blood and activate blood, but can also provide beneficial effects against arteriosclerosis, bacteriostasis and anoxia. Angelica can also regulate the immune function of the body [15]. RA patients have clinical manifestations of qi and blood deficiency throughout the development of the disease [16]. DBD, as the representative formula of Invigorating Qi and promoting blood circulation, is mainly used to treat the syndrome of fatigue and internal injury, Qi weakness, and blood deficiency. Couples of studies also have investigated the effect of the main active ingredients of DBD on RA. Studies have shown that astragaloside IV has anti-arthritis effects and can prevent joint inflammation and cartilage destruction [17]. The results of Doss HM et al. showed that ferulic acid exerts anti-osteoclast differentiation and bone erosion by inhibiting RANKL-dependent NF-κB signaling pathway to treat RA [18]. Additionally, Calycosin was reported to be capable of activating p62/Nrf2-linked heme oxygenase 1 in RA synovial fibroblast [19]. However, it is important to note that, to date, few studies have directly investigated the effect of DBD on RA. Therefore, we selected DBD as a treatment for RA.

The main characteristics of RA are the proliferation of synovial tissue and the destruction of joint tissue. The main cause of these symptoms is the imbalance of proliferation and apoptosis of the FLS in RA patients; this results in “tumor like” proliferation [20]. FLS are important modulators of synovial inflammation in RA progression [21, 22]. FLS could secrete pro-inflammatory cytokines and mediators, which modulate growth factor expressions and trigger more FLS activations as feedback [23–27]. Previous research has shown that TNF-α can stimulate synovial cells, fibroblasts, osteoclasts, and chondrocytes, to produce matrix metalloproteinases; these substances can damage cartilage and lead to the continuous aggravation of synovial inflammation in RA patients, thus gradually destroying cartilage and bone tissue [28]. IL-6 is one of the factors responsible for the pathogenesis of RA and is involved in the early activation of early B cells and T cells in the early process of RA and then throughout the entire process of RA. TNF-α can promote the production of IL-6 in synovial cells; IL-6 can also enhance the pro-inflammatory effects of TNF-α and other cytokines [29]. IL-1β, as a pro-inflammatory factor, is produced by activated macrophages and is one of the important mediators involved in the inflammatory response; thus, this factor is related to cell proliferation, differentiation, and apoptosis [30]. IL-4 is produced by CD4^+^ T cells that are stimulated by antigens and are the generation and differentiation factor for many hematopoietic progenitor cells. As a bone absorption inhibitor, IL-4 plays an important role in regulating bone metabolism [31]. IL-1β, IL-6, IL-17 and TNF-α play key roles in the pathophysiological process of RA [32, 33]. Under the stimulation of IL-10 or TGF-β, macrophages can differentiate into 2C subtypes of type 2 macrophages; the main functions of these macrophages are to inhibit the inflammatory response and promote tissue repair. IL-10 can also regulate immune response and inflammatory response but also plays a negative role in the local inflammatory response [34, 35]. Inhibition of pro-inflammatory cytokines can further weaken the inflammatory response and reduce cartilage damage [36]. Our current results showed that the proliferative activity of HFLS-RA cells in vitro increased significantly following TNF-α stimulation. Following intervention with the serum-containing DBD, the proliferative activity of HFLS-RA cells decreased gradually and the inhibitory effect of serum-containing DBD in the medium dose group was better. TNF-α can promote the secretion of IL-6 by synovial cells, thus aggravating inflammation and the destruction of cartilage. The levels of TNF-α, IL-1β, IL-6, and IL-10 in HFLS-RA cells induced by TNF-α were significantly decreased but significantly increased after intervention with the drug-containing serum of DBD, thus indicating that the drug-containing serum of DBD can control the inflammatory response of disease by inhibiting inflammatory cytokines.

The Wnt signaling pathway, as a highly conserved signaling pathway throughout evolution, is involved in a wide range of biological processes, including growth, development, metabolism and stem cell maintenance [37]. Some previous studies have shown that the Wnt signaling pathway is closely related to the pathogenesis of RA. Previous studies have demonstrated that there is a high level of β-catenin in the FLS of RA patients and that high levels of β-catenin can maintain the stable activation of the FLS, thus causing the expression of inflammatory factors, such as TNF-α, IL, and mediate the inflammatory response of RA [38]. The over-activation of the Wnt signaling pathway and the abnormal expression of β-catenin plays an important role in bone destruction in RA and can cause serious damage to the osteogenic and osteoclastic balance required for normal bone reconstruction, thus causing gradual bone destruction. Therefore, inhibition of the Wnt/β-catenin signaling pathway is useful in alleviating the occurrence and development of arthritis [39]. SFRP4 and c-myc are important upstream and downstream effector genes of Wnt signaling pathway. When RA occurs, the expression of SFRP4 is down-regulated, the expression of β-catenin and c-myc are up-regulated, and the synovial hyperplasia becomes abnormal [40].

The Wnt signaling pathway plays an important role in the abnormal proliferation of the synovium in RA. Therefore, we investigated the role of DBD in the regulation of the Wnt signaling pathway in the synovium of the rat model of CIA and investigated the mechanisms involved. Our results showed that the swelling and hyperemia of the synovium, along with the proliferation of synovium cells, in rats treated with low, middle and high doses of DBD were improved by varying degrees; moreover, there was no obvious inflammatory cell infiltration, vascular proliferation, or fibrosis. The levels of IL-1 and IL-6 in the serum of rats in the tripterygium glycoside group and DBD medium and high dose group were significantly decreased, while the serum levels of IL-4 and IL-10 were significantly increased (*P* < 0.05). The expression level of SFRP4 in the Wnt pathway was high prior to RA. However, during the development of RA, the expression levels of SFRP4 were down-regulated, while the expression of β-Catenin and c-myc in the Wnt pathway were up-regulated; this led to the abnormal proliferation of the synovium. After the oral administration of DBD by gavage, we found that DBD could up-regulate SFRP4 (which was expressed at low levels in the synovium of rats with arthritis) and that the protein levels of β-catenin and c-myc (downstream of the Wnt pathway) decreased significantly, thus inhibiting the abnormal proliferation in the synovium of rats with arthritis.

## Conclusion

In conclusion, our data suggest that DBD can inhibit the development of RA and that it may be possible to inhibit the inflammatory response of inflammatory cytokines to control disease and delay the development of RA by inhibiting the Wnt signal transduction pathway with SFRP4 as the main target. These results reveal the pharmacologic mechanism for RA further research.

## Data Availability

We state that the data will not be shared since all the raw data are present in the figures included in the article.

## Abbreviations

DBD: Danggui Buxue Decoction
TNF: Tumor necrosis factor
HFLS-RA: Human fibroblast-like synoviocyte of rheumatoid arthritis
CIA: Collagen-induced arthritis
RA: Rheumatoid arthritis
TCM: Traditional Chinese medicine
MTT: Thiazolyl blue tetrazolium bromide
CCK-8: Cell Counting Kit-8
DMSO: Dimethyl sulfoxide
EDTA: Ethylenediaminetetraacetic acid
OD: Optical density
AI: Arthritis index
HE: Hematoxylin–eosin
AVOVA: Analysis of variance
FLS: Fibroblast-like synoviocyte
IL: Interleukin
SFRPS: Secretory frizzled-related proteins
